# Supplementary material for: Transcriptome Analysis Reveals the Molecular Mechanism of PLIN1 in Goose Hierarchical and Pre-Hierarchical Follicle Granulosa Cells
Source: Animals (Basel). 2025 Jan 20;15(2):284. doi: 10.3390/ani15020284 (PMC11761271; doi:10.3390/ani15020284)
Supplement: Supplementary file 1 [file animals-15-00284-s001.zip › files S1.docx]

ATGGTGGCGGAGGGGCCATTTTTGCAGCCACCCGTCCCAAATTTGGGTGGCCTCGGGGAGCTGTTTGCCCAGTTTCCACAGCAACCCGTGGCTCCGAACCCAGCTGGGAGGTGGGGCCTGGGGGTGGTAGTGCCCGGGGCACCGCGCACCCCGGCACAGGACCCGCACGCAGGGACGGCGCTGCCACCACCTCCTGCTCTGGACAGGCAGACCCTCGGCGGACGTGTCGCGATGACGGCCAAGAAGAATCAGACCCTGCAGGATGGAAGTGCCAAGGAGAACGTGCTGCAGCGGGTCCTGCAGCTGCCGGTGGTCAGCTCGACCTGCGAAAGCCTCCAGCGGACCTACGCCAGCACCAAGGAGGCCCACCCGCTCATGGCCTCCGTGTGCGAGGTCTACGAGCGGGGCGTGCAGGGCGCCAGCGCCTTGGCCTTGTGGAGCGTGGAGCCCGTGGTGCGCAGGCTGGAGCCCCAGTTCACTGTAGCCAACAACTTGGCCTGCCGGGGCTTGGACCACCTGGAGGAGAAGATCCCTGCCCTGCAGTACCCCGTCGACAAGCTCGCCTCCGAACTGAAGGACACCATCTCCACCCCCATCCAAAGTGCCAAAAGCACCATTGGCAACTCCATGGACAAGATCCTGGGGCTGGCGGTGGAGGGCTATGAGGCGACCAAGAGCACCGTGGAGACCACGGCCAGGTACACGAGGAGCAACTCGGTGAGCCAGATGGCGGCCGCCGGCGTCGACACGGCGCTGGGCGGGCTGGAGAAGCTGATGGAGTACCTGCTGCCAGAAGAGGACGAGGAAGCAGATCAGAAGCCCAAAGAGACTCGTGGGGCAGCAGCAAAGGTCTCCCAGAGGAAGCCCAGCGCTCCCAGAGCTCCCAGTGCTCCCAGTGCTCCCAGTGCTCCCAGCACTCCCAGTGCTCCCAGCACCCTGGGCCGGATCGGAGCCCTGGTCAGCACCGTCTCCCACCGCGCTTACCAGCAAACCACCCAGAGCCTCCAGCGTGCCAAAACCAAAGGGCAGGAGCTGGTCACCTGGATCCCCATCGTGGGCAGCCTGGCCAAGCCGAGCGCACCCCAGGCACCACAAAGCCACGGCGAGGGGCAGGGCACTGCGGGCGGCTGGCTGAGCCGGCGGCAGAGCAAGGTGCCGGAGCAGAAGCAGGAGAAAGCGGGGAAGAAGGAGGAGAGCCACGCCAGTGAGGCAGGGGACGGCCCCGGGCTGGTGGGCAGCGTGGCCCACAACCTGCAGAGCGCCTGCGCCTCCGGCATCTCCAGCGTGAAGAAGGTCCCCGCCGTGGCCTGGGACGCGGCCGAGGGGTTGATCCTGTTCACCCCCCGCAGGCTGTCCAGGGCCATGGAGACGGTGGATGCACTGGGGGGGACCCTCGTCAGCGCCCCCAAGCATCTGCTGGGCACCCTGTACAGCTACGTGCCGCTCCGCAGGCAGTCGGTGAAGGACGAGGTGGCGGCCGGGGGCAGCAAGGCCGGCCCTGAGACGGAGCAGAAGGAGGAGGACGGCAAGCCCGCCGCTGCCCCCGCCGAGGACAAGTCCCAGCTGAGGGGCGACTGGCGGCTGTACCGCGGCCACCACCCGCTGTCCTTCCTGGGCCTGGAGGACCCCCTCTTCGTGCGCCACAACCTCTACCGCAGCCCGGCCTTCGAGCCCGAGTGCCCCCTGCCGCGGAAATCCGCCTTCGCCCCCTACAACCGGCGGGTGAGCGAGGGCTCCTACCGCTTCGGCCCCGAGGCCATGTACAGCCGGGCCTACTACGCCAACCTCTACAGCCCGGCCTTCAAGAAGGACTGA
