# Supplementary material for: Transcriptome Analysis Reveals the Molecular Mechanism of PLIN1 in Goose Hierarchical and Pre-Hierarchical Follicle Granulosa Cells
Source: Animals (Basel). 2025 Jan 20;15(2):284. doi: 10.3390/ani15020284 (PMC11761271; doi:10.3390/ani15020284)
Supplement: Supplementary file 1 [file animals-15-00284-s001.zip › Table S3.docx]

Table S3. Functional description of key genes and display of enriched pathways.

| Gene | Gene description | KEGG pathway/GO Term | Gene function |
| --- | --- | --- | --- |
| PPARG | Peroxisome Proliferator Activated Receptor Gamma | PPAR signaling pathway | Regulate adipocyte differentiation, lipid storage, and insulin sensitivity, and positively regulate the transcription of PLIN1 |
| MGLL | Monoglyceride Lipase | Metabolic pathway | Control the level of FFA in cells and play an important role in lipid metabolism |
| PTEN | Phosphatase And Tensin Homolog | P53 signaling pathway | Promote granulosa cell proliferation, steroid production, and lipid accumulation, regulate follicular development |
| BAMBI | BMP And Activin Membrane Bound Inhibitor | TGF-beta signaling pathway | Regulating the proliferation of granulosa cells and the synthesis of steroid hormones, promoting signal transduction of TGF - β steroid production in granulosa cells |
| JUN | Jun Proto-Oncogene, AP-1 Transcription Factor Subunit | Apoptosis | Regulating cell proliferation, migration, and apoptosis, indirectly regulating steroid hormone biosynthesis and endoplasmic reticulum stress |
| FST | Follistatin,activin-Binding protein | TGF-beta signaling pathway | Inhibiting oxidative stress by neutralizing ROS to prevent cell apoptosis |
| ACSF3 | Acyl-CoA Synthetase Family Member 3 | Fatty acid metabolism | A mitochondrial enzyme that plays a crucial metabolic editing function, allowing highly metabolically active cells to continue breathing |
| ACSL4 | Acyl-CoA Synthetase Long Chain Family Member 4 | Fatty acid biosynthesis | Regulating cell lipid droplet deposition |
